# The relationship between women’s personality traits and addiction to social networking sites on the example of Facebook

**DOI:** 10.1192/j.eurpsy.2023.349

**Published:** 2023-07-19

**Authors:** A. M. Cybulska, K. Rachubińska, D. Schneider-Matyka, S. Grochans, E. Grochans

**Affiliations:** ^1^Department of Nursing; ^2^Department of Clinical Nursing, Pomeranian Medical University in Szczecin, Szczecin, Poland

## Abstract

**Introduction:**

Social network addicts may differ significantly from non-addicts in terms of personality traits, such as agreeableness, neuroticism, and conscientiousness. Addicts may be prone to negative emotions and unpleasant experiences, which may be associated with a higher level of neuroticism. Addicts often fail to cope in the real world, often experience negative emotions, quickly give up their goals, see themselves and others in a negative light, and escape into virtual reality. The virtual world is devoid of the anxiety that an individual faces in real life interactions, hence the tendency for addiction among people with higher levels of neuroticism. The primary cause of Internet Addiction may be innate temperamental traits (i.e., impulsiveness of behavior and impulsiveness of decisions) that influence the susceptibility to functional and chemical addictions. According to the concept of addictive personality, people who have this problem are prone to addiction as such, regardless of what they are addicted to.

**Objectives:**

The purpose of this study was to generally assess the degree of women’s dependence on social networking sites on the example of Facebook, taking into account personality traits according to the five-factor model of personality, the so-called Big Five by Costa and McCrae

**Methods:**

The study included 556 women. This survey-based study was carried out using the questionnaire technique. The following research tools were used to analyze behavioral addictions in adult women: the Bergen Face- book Addiction Scale (BFAS), the NEO Five-Factor Inventory (NEO FFI) and the author’s questionnaire.

**Results:**

Among the surveyed women, 69.6% were average Facebook users, of whom 16.4% had scores indicating possible Facebook addiction, and 14.0% had scores indicating addiction. The higher the neuroticism, the more serious the Facebook addiction (r = 0.26; p < 0.001; R2 = 6.7%). A weak negative correlation was obtained for the agreeableness subscale e (r = -0.08; p < 0.05; R2 = 7.2%). A weak negative correlation was also obtained for the subscale of conscientiousness (r = -0.16; p < 0.001; R2 = 2.6%). There was no statistically significant correlation between the log10 score obtained on the BFAS and the score on the NEO-FFI subscales of extraversion (r = 0.04; p = 0.40) and openness to experience (r = 0.04; p = 0.30).

**Conclusions:**

The personality types of the studied women indicated relationships in terms of behavioral addictions. Women characterized by neuroticism showed stronger addiction to Facebook. Women characterized by high conscientiousness were at lower risk of behavioral addictions, while agreeableness as a personality trait significantly protected the surveyed women against Facebook addiction.

**Disclosure of Interest:**

None Declared

